# Supplementary material for: Remembrance of things practiced with fast and slow learning in cortical and subcortical pathways
Source: Nat Commun. 2020 Dec 23;11:6441. doi: 10.1038/s41467-020-19788-5 (PMC7758336; doi:10.1038/s41467-020-19788-5)
Supplement: Supplementary file 1 — Supplementary Information [file 41467_2020_19788_MOESM1_ESM.pdf]

Supplemental Information for "Remembrance of things practiced with fast and slow learning in cortical and subcortical pathways"

James M. Murray and G. Sean Escola

Supplementary Note 1: The perceptron forgetting curve

As described in the main text, we wish to train a perceptron having $N_x = N$ inputs and subject to the update rule (1) to map P random input patterns onto randomly chosen binary outputs. The question that we seek to answer is, after P patterns have been trained using the update rule in (1), what will be the probability of misclassification if we then test the output produced by a particular pattern ν without any further learning? Clearly, the most recently learned patterns are likely to produce correct outputs, while those learned long in the past are more likely to produce errors due to accumulated changes in the weights $\mathbf{w}$ during subsequent learning. In general, the probability of an error when testing on pattern ν is, using the Heaviside step function $\Theta(\cdot)$, given by

$$p(z^\nu \neq 1) = \int d\mathbf{x}^\nu p(\mathbf{x}^\nu) \int d\mathbf{w}^\nu p(\mathbf{w}^\nu) \int d\mathbf{w}^P \Big[p(\mathbf{w}^P|\mathbf{w}^\nu + [1 - \mathbf{w}^\nu \cdot \mathbf{x}^\nu]\mathbf{x}^\nu/N)\Theta(-\mathbf{w}^P \cdot \mathbf{x}^\nu)\Theta(1 - \mathbf{w}^\nu \cdot \mathbf{x}^\nu) + p(\mathbf{w}^P|\mathbf{w}^\nu)\Theta(-\mathbf{w}^P \cdot \mathbf{x}^\nu)\Theta(\mathbf{w}^\nu \cdot \mathbf{x}^\nu - 1)\Big]. \tag{6}$$

In this equation, we have, without loss of generality, redefined $\mathbf{x}^\mu \to \hat{z}^\mu \mathbf{x}^\mu$, so that the target output becomes $z^\mu = 1$ for every pattern. We have also set the classification margin $\kappa = 1$, which amounts to a choice for scaling the overall magnitude $|\mathbf{w}|$. In this equation, the weight vector just before training pattern ν is assumed to come from a distribution $p(\mathbf{w}^\nu)$, which we shall derive below. The first line in (6) counts the cases in which the classification using this weight vector is initially incorrect or correct with margin less than $\kappa = 1$ (so $1 - \mathbf{w}^\nu \cdot \mathbf{x} > 0$) and in which, after making the initial update $\mathbf{w}^\nu \to \mathbf{w}^\nu + \Delta\mathbf{w}^\nu$, the weight vector evolves through $P - \nu$ successive updates into the final weight vector $\mathbf{w}^P$, which leads to incorrect classification when pattern ν is again tested ($-\mathbf{w}^P \cdot \mathbf{x}^\nu > 0$). Similarly, the second line of (6) counts the cases in which the classification is initially correct with a sufficiently large margin and in which, after making successive weight updates, the final weight vector $\mathbf{w}^P$ again leads to incorrect classification.

At this stage, the probability distributions $p(\mathbf{w}^\nu)$ and $p(\mathbf{w}^P|\mathbf{w}^\nu)$ in (6) are unknown. For the latter distribution, however, we can track its evolution step by step using the update rule (1). Because $\mathbf{x}$ is a random variable, we first seek to find the distribution $p(\Delta\mathbf{w}|\mathbf{w})$ by averaging over $\mathbf{x}$. In fact, it will be sufficient just to calculate the first two moments of this distribution. Let $\mathbf{x} = \mathbf{x}^\parallel + \mathbf{x}^\perp$, where $\mathbf{x}^\parallel$ is the component along $\mathbf{w}$, and the index ν has been dropped for simplicity. In this case the weight update (1), in the case where an update occurs, can be written as

$$\Delta\mathbf{w} = \frac{1}{N}(1 - |\mathbf{w}|x^\parallel)(x^\parallel \mathbf{w}/|\mathbf{w}| + \mathbf{x}^\perp). \tag{7}$$

Using (7), in the case where a weight update occurs, the first moment is given by

$$\begin{aligned} \mu_i(\mathbf{w}) &\equiv \langle \Delta w_i \rangle_\mathbf{x} \\ &= \frac{1}{N} \int \frac{dx^\parallel}{\sqrt{2\pi}} e^{-(x^\parallel)^2/2} \int \frac{d\mathbf{x}^\perp}{(2\pi)^{(N-1)/2}} e^{-|\mathbf{x}^\perp|^2/2} (1 - |\mathbf{w}|x^\parallel)(x^\parallel w_i/|\mathbf{w}| + x_i^\perp) \\ &= -\frac{1}{N} w_i \int \frac{dx^\parallel}{\sqrt{2\pi}} e^{-(x^\parallel)^2/2} (x^\parallel)^2 \\ &= -\frac{1}{N} w_i. \end{aligned} \tag{8}$$

Similarly, the second moment of the distribution is

$$\begin{aligned}
\Sigma_{ij}(\mathbf{w}) &\equiv \langle \Delta w_i \Delta w_j \rangle_{\mathbf{x}} - \mu_i(\mathbf{w})\mu_j(\mathbf{w}) \\
&= \frac{1}{N^2}\int \frac{dx^{\parallel}}{\sqrt{2\pi}} e^{-(x^{\parallel})^2/2} \int \frac{d\mathbf{x}^{\perp}}{(2\pi)^{(N-1)/2}} e^{-|\mathbf{x}^{\perp}|^2/2} (1-|\mathbf{w}|x^{\parallel})^2 \left(\frac{x^{\parallel} w_i}{|\mathbf{w}|} + x_i^{\perp}\right)\left(\frac{x^{\parallel} w_j}{|\mathbf{w}|} + x_j^{\perp}\right) - \mu_i(\mathbf{w})\mu_j(\mathbf{w}) \\
&= \frac{1}{N^2}\int \frac{dx^{\parallel}}{\sqrt{2\pi}} e^{-(x^{\parallel})^2/2} \left[1+|\mathbf{w}|^2 (x^{\parallel})^2\right]\left[(x^{\parallel})^2 \frac{w_i w_j}{|\mathbf{w}|^2} + \delta_{ij}\right] - \mu_i(\mathbf{w})\mu_j(\mathbf{w}) \\
&= \frac{2}{N^2}\left[g(|\mathbf{w}|)\delta_{ij} + h(|\mathbf{w}|) w_i w_j\right]
\end{aligned} \tag{9}$$

where

$$\begin{aligned}
g(|\mathbf{w}|) &\equiv \frac{1+|\mathbf{w}|^2}{2} \\
h(|\mathbf{w}|) &\equiv \frac{2|\mathbf{w}|^2+1}{2|\mathbf{w}|^2}.
\end{aligned} \tag{10}$$

Together, (8)-(10) describe a drift-diffusion process. If we neglect higher-order moments, then the single-step probability distribution is given by

$$p(\Delta\mathbf{w}|\mathbf{w}) = q\mathcal{N}(\boldsymbol{\mu}(\mathbf{w}), \boldsymbol{\Sigma}(\mathbf{w})) + (1-q)\delta(\Delta\mathbf{w}), \tag{11}$$

where q (to be calculated below) is the probability that a weight update occurs in a given step, and $\mathcal{N}(\cdot,\cdot)$ is the multinormal distribution. The time evolution of the probability distribution of the weights is then given by the Fokker-Planck equation (Risken, 1996):

$$\frac{dp(\mathbf{w}^{\tau})}{d\tau} = -\sum_i \frac{\partial}{\partial w_i^{\tau}}\left[\mu_i(\mathbf{w}^{\tau})p(\mathbf{w}^{\tau})\right] + \frac{1}{2}\sum_{ij}\frac{\partial^2}{\partial w_i^{\tau}\partial w_j^{\tau}}\left[\Sigma_{ij}(\mathbf{w}^{\tau})p(\mathbf{w}^{\tau})\right], \tag{12}$$

where the initial condition from (6) is either $p(\mathbf{w}) = \delta(\mathbf{w}-\mathbf{w}^{\nu})$ or $p(\mathbf{w}) = \delta(\mathbf{w}-\mathbf{w}^{\nu}-\Delta\mathbf{w}^{\nu})$, and $\tau \equiv q(P-\nu)$ is the effective time variable.

Because the coefficients in (12) depend on $|\mathbf{w}^{\tau}|$, the full solution is not known in general. However, it is straightforward to calculate the time evolution of the moments of $p(\mathbf{w}^{\tau})$ by multiplying both sides by powers of $\mathbf{w}^{\tau}$ and using integration by parts (Risken, 1996). Denoting the first two moments (not to be confused with the moments of $\Delta\mathbf{w}$ defined above) as

$$\begin{aligned}
\tilde{\mu}_i(\tau) &\equiv \langle w_i \rangle \\
\tilde{\Sigma}_{ij}(\tau) &\equiv \langle w_i w_j \rangle - \langle w_i \rangle \langle w_j \rangle,
\end{aligned} \tag{13}$$

the time evolution is given by

$$\begin{aligned}
\dot{\tilde{\mu}}_i &= -\frac{\tilde{\mu}_i}{N} \\
\dot{\tilde{\Sigma}}_{ij} &= -\frac{2}{N}\left(1-\frac{h}{N}\right)\tilde{\Sigma}_{ij} + \frac{2h}{N^2}\tilde{\mu}_i\tilde{\mu}_j + \frac{2g}{N^2}\delta_{ij}.
\end{aligned} \tag{14}$$

In the $N \to \infty$ limit, we can assume (to be checked below) that $|\mathbf{w}^{\tau}|$ is constant. In this case, (14) has the solutions

$$\begin{aligned}
\tilde{\mu}_i(\tau) &= -\frac{1}{N} w_i^{\nu} e^{-\tau/N} \\
\tilde{\Sigma}_{ij}(\tau) &\overset{(N\to\infty)}{=} \frac{2h\tau}{N^2} e^{-2\tau/N} w_i^{\nu} w_j^{\nu} + \delta_{ij}\frac{g}{N}\left(1-e^{-2\tau/N}\right),
\end{aligned} \tag{15}$$

where terms $\sim O(1/N^2)$ in the second equation have been dropped in the large-N limit (while the first term is kept because τ may be $\sim O(N)$).

With (15), the solution to the Fokker-Planck equation (12) when just the first two moments are kept is

$$\begin{aligned} p(\mathbf{w}^P|\mathbf{w}^\nu) =& \frac{1}{[2\pi g(1-\gamma^2)/N]^{N/2}} \\ &\times \exp\left(-\frac{N}{2g(1-\gamma^2)}\left[\mathbf{w}^P-\gamma\mathbf{w}^\nu\right]\cdot\left[1-\frac{2h\gamma^2 q(P-\nu)}{Ng(1-\gamma^2)}\mathbf{w}^\nu\otimes\mathbf{w}^\nu\right]\cdot\left[\mathbf{w}^P-\gamma\mathbf{w}^\nu\right]\right), \end{aligned} \tag{16}$$

where "$\otimes$" denotes the outer product (i.e. $[\mathbf{w}\otimes\mathbf{w}]_{ij}=w_i w_j$), and we have defined

$$\gamma \equiv e^{-q(P-\nu)/N}. \tag{17}$$

In the $N\to\infty$ limit, the anisotropic term can be ignored, giving

$$p(\mathbf{w}^P|\mathbf{w}^\nu) \overset{(N\to\infty)}{=} \frac{1}{[2\pi g(1-\gamma^2)/N]^{N/2}}\exp\left(-\frac{N}{2g(1-\gamma^2)}\left[\mathbf{w}^P-\gamma\mathbf{w}^\nu\right]^2\right), \tag{18}$$

which is the probability density evolution corresponding to an Ornstein-Uhlenbeck stochastic process (Risken, 1996). After a long time, $\gamma\to 0$ and $p(\mathbf{w}^P|\mathbf{w}^\nu)$ from (16) approaches the steady-state distribution

$$p(\mathbf{w}) \equiv \frac{1}{[2\pi g/N]^{N/2}}\exp\left(-\frac{N}{2g}|\mathbf{w}|^2\right). \tag{19}$$

Thus, the deterministic update rule (1) leads to a bounded steady-state weight distribution $p(\mathbf{w})$ after a large number of classifications have been learned. This differs somewhat from previous models of sequential learning with random synaptic weight updates, such a bounded distribution as $P/N\to\infty$ was achieved either by requiring that the synaptic weights should be bounded (Fusi, 2007) or that that they should decay slightly at each step (Benna, 2016).

Given the steady-state distribution (19), we can assume that $\hat{w}\equiv|\mathbf{w}|$ is constant in the large-N limit and let $\hat{g}\equiv g(\hat{w})$. Then, with $g=\hat{g}$, (19) can be used to calculate the variance of $\mathbf{w}$, leading to the self-consistent equation $\hat{w}^2=\hat{g}$, which, using the definition of $g(|\mathbf{w}|)$ from (10), has the solution $\hat{w}^2=\hat{g}=1$. This is close to but differs somewhat from the steady-state norm found in numerical simulations, from which $\hat{w}\simeq 1.19$. The reason for this is presumably because of the decision to approximate $p(\Delta\mathbf{w}|\mathbf{w})$ using only the first two moments of the distribution in (12). In general, the higher-order moments do not vanish, and these will contribute higher-order derivative terms in the Fokker-Planck equation (13). In turn, such terms will lead to nonvanishing higher-order moments in the distribution $p(\mathbf{w})$, beyond the two that were calculated in (13)-(15). Presumably, it is these higher-order terms which cause the discrepancy between the simulated result and the self-consistent calculation. In the theoretical curves shown in the Results section, we use the value of $\hat{w}$ obtained from simulations. Using (19), we can also calculate the probability of making a weight update in a given step, which is given by

$$\begin{aligned} q &= \int\frac{d\mathbf{w}}{(2\pi\hat{w}^2/N)^{N/2}}\int\frac{d\mathbf{x}}{(2\pi)^{N/2}}e^{-|\mathbf{x}|^2/2}e^{-N|\mathbf{w}|^2/2\hat{w}^2}\Theta(1-\mathbf{w}\cdot\mathbf{x}) \\ &= \frac{1}{2}\mathrm{erfc}\left(-\frac{1}{\sqrt{2}\hat{w}}\right) \end{aligned} \tag{20}$$

and evaluates to $q\simeq 0.798$.

With the preceding points in mind, and making use of the probability distributions (18) and (19), we can proceed to evaluate the integrals in (6) to obtain the probability of incorrect classification when testing

pattern ν. In order to factorize the arguments of the Heaviside step functions, we make use of the following identity:

$$\begin{aligned}\Theta(\rho) &= \int_0^\infty du\ \delta(u-\rho) \\ &= \int_0^\infty du \int_{-\infty}^{\infty} \frac{dv}{2\pi} e^{iv(u-\rho)}.\end{aligned} \tag{21}$$

with this trick, (6) becomes

$$p(z^\nu \neq 1) = I_1 + I_2, \tag{22}$$

where

$$\begin{aligned}I_1 = &\int_0^\infty du \int_0^\infty du' \int_{-\infty}^{\infty} \frac{dv}{2\pi} \int_{-\infty}^{\infty} \frac{dv'}{2\pi} e^{i(uv+u'v')} \int \frac{d\mathbf{x}}{(2\pi)^{N/2}} e^{-\mathbf{x}^2/2} \\ &\times \int \frac{d\mathbf{w}^\nu}{(2\pi\hat{g}/N)^{N/2}} e^{-N|\mathbf{w}^\nu|^2/2\hat{g}} \int \frac{d\mathbf{w}^P}{[2\pi(1-\gamma^2)\hat{g}/N]^{N/2}} e^{i[v\mathbf{w}^P\cdot\mathbf{x}+v'(\mathbf{w}^\nu\cdot\mathbf{x}-1)]} \\ &\times \exp\left(\frac{-N\left[\mathbf{w}^P - \gamma(\mathbf{w}^\nu + [1-\mathbf{w}^\nu\cdot\mathbf{x}]\mathbf{x}/N)\right]^2}{2\hat{g}(1-\gamma^2)}\right)\end{aligned} \tag{23}$$

and

$$\begin{aligned}I_2 = &\int_0^\infty du \int_0^\infty du' \int_{-\infty}^{\infty} \frac{dv}{2\pi} \int_{-\infty}^{\infty} \frac{dv'}{2\pi} e^{i(uv+u'v')} \int \frac{d\mathbf{x}}{(2\pi)^{N/2}} e^{-\mathbf{x}^2/2} \\ &\times \int \frac{d\mathbf{w}^\nu}{(2\pi\hat{g}/N)^{N/2}} e^{-N|\mathbf{w}^\nu|^2/2\hat{g}} \int \frac{d\mathbf{w}^P}{[2\pi(1-\gamma^2)\hat{g}/N]^{N/2}} e^{i[v\mathbf{w}^P\cdot\mathbf{x}+v'(1-\mathbf{w}^\nu\cdot\mathbf{x})]} \\ &\times \exp\left(\frac{-N\left[\mathbf{w}^P - \gamma\mathbf{w}^\nu\right]^2}{2\hat{g}(1-\gamma^2)}\right)\end{aligned} \tag{24}$$

Beginning with I_1, the integral over $\mathbf{w}^P$ can be performed, which, after simplification and using $\mathbf{x}^2 = N$ in the $N \to \infty$ limit, leads to

$$\begin{aligned}I_1 = &\int_0^\infty du \int_0^\infty du' \int_{-\infty}^{\infty} \frac{dv}{2\pi} \int_{-\infty}^{\infty} \frac{dv'}{2\pi} e^{i[(u+\gamma)v+(u'-1)v']} \int \frac{d\mathbf{x}}{(2\pi)^{N/2}} e^{-\mathbf{x}^2/2} \\ &\times \int \frac{d\mathbf{w}^\nu}{(2\pi\hat{g}/N)^{N/2}} \exp\left(-\frac{N}{2\hat{g}}|\mathbf{w}^\nu|^2 + iv'\mathbf{w}^\nu\cdot\mathbf{x} - \frac{\hat{g}(1-\gamma^2)}{2}v^2\right).\end{aligned} \tag{25}$$

Performing the integrals over $\mathbf{w}^\nu$ and $\mathbf{x}$ then leads to

$$\begin{aligned}I_1 &= \int_0^\infty du \int_0^\infty du' \int_{-\infty}^{\infty} \frac{dv}{2\pi} \int_{-\infty}^{\infty} \frac{dv'}{2\pi} e^{i[(u+\gamma)v+(u'-1)v']-\hat{g}(1-\gamma^2)v^2/2} \left(1+\frac{\hat{g}v'^2}{N}\right)^{-N/2} \\ &\overset{(N\to\infty)}{=} \int_0^\infty du \int_0^\infty du' \int_{-\infty}^{\infty} \frac{dv}{2\pi} \int_{-\infty}^{\infty} \frac{dv'}{2\pi} e^{i[(u+\gamma)v+(u'-1)v']-\hat{g}(1-\gamma^2)v^2/2-\hat{g}v'^2/2}.\end{aligned} \tag{26}$$

Finally, the integrals over v and v' can be performed exactly, then those over u and u' can be evaluated using the complementary error function, yielding the final result

$$I_1 = \frac{1}{4}\mathrm{erfc}\left(\frac{\gamma}{\sqrt{2\hat{g}(1-\gamma^2)}}\right)\mathrm{erfc}\left(-\frac{1}{\sqrt{2\hat{g}}}\right). \tag{27}$$

In a similar manner, the integrals in (24) can be evaluated to get

$$\begin{aligned}
I_2 &= \int_0^\infty du \int_0^\infty du' \int_{-\infty}^\infty \frac{dv}{2\pi} \int_{-\infty}^\infty \frac{dv'}{2\pi} e^{i[uv+(u'+1)v']} \int \frac{d\mathbf{x}}{(2\pi)^{N/2}} e^{-\mathbf{x}^2/2} \\
&\quad \times \int \frac{d\mathbf{w}^\nu}{(2\pi\hat{g}/N)^{N/2}} \exp\left(-\frac{N}{2\hat{g}}|\mathbf{w}^\nu|^2 + [\gamma v - v']\mathbf{w}^\nu \cdot \mathbf{x} - \frac{\hat{g}(1-\gamma^2)}{2N} v^2 \mathbf{x}^2\right) \\
&= \int_0^\infty du \int_0^\infty du' \int_{-\infty}^\infty \frac{dv}{2\pi} \int_{-\infty}^\infty \frac{dv'}{2\pi} e^{i[uv+(u'+1)v']} \left[1 + \frac{\hat{g}}{N}(v^2 + v'^2 - 2\gamma v v')\right]^{-N/2} \\
&\overset{(N\to\infty)}{=} \int_0^\infty du \int_0^\infty du' \int_{-\infty}^\infty \frac{dv}{2\pi} \int_{-\infty}^\infty \frac{dv'}{2\pi} \exp\left(i[uv + (u'+1)v'] - \frac{\hat{g}}{2}(v^2 + v'^2 - 2\gamma v v')\right).
\end{aligned} \tag{28}$$

After performing the integrals over v, v', and u', then changing variables for the u integral, the final result is

$$I_2 = \frac{1}{\sqrt{8\pi}} \int_0^\infty dr\ e^{-r^2/2} \text{erfc}\left(\frac{\gamma r + 1/\sqrt{\hat{g}}}{\sqrt{2(1-\gamma^2)}}\right), \tag{29}$$

where the final integral in this case must be performed numerically.

As a check, we can evaluate these results in their extreme limits. In the case $\gamma = 1$, which corresponds to testing the most recently learned pattern, we have $I_1 \sim I_2 \sim \text{erfc}(\infty) \to 0$, so that there is perfect classification for very recently learned patterns. In the opposite limit of $\gamma = 0$, which corresponds to testing patterns learned in the distant past, we obtain $I_1 + I_2 = \frac{1}{4}[\text{erfc}(-1/\sqrt{\hat{g}}) + \text{erfc}(1/\sqrt{\hat{g}})] = \frac{1}{2}$, which means that very old patterns are completely overwritten and so are classified at chance level.

Supplementary Note 2: The two-pathway forgetting curve

Let us introduce a second source of input to the downstream units, so that $z^\mu = \phi(\mathbf{w} \cdot \mathbf{x}^\mu + \mathbf{v} \cdot \mathbf{y}^\mu)$, where $x_i^\mu, y_i^\mu \sim \mathcal{N}(0,1)$. Though it is not necessary, we will assume for notational simplicity that the numbers of units in the two input layers are the same, so that $N_x = N_y = N$. The weights $\mathbf{w}$ are again trained using supervised learning:

$$\Delta\mathbf{w}^\mu = \begin{cases} (\kappa\hat{z}^\mu - \mathbf{w}^\mu \cdot \mathbf{x}^\mu - \mathbf{v}^\mu \cdot \mathbf{y}^\mu)\mathbf{x}^\mu/N, & (\mathbf{w}^\mu \cdot \mathbf{x}^\mu + \mathbf{v}^\mu \cdot \mathbf{y}^\mu)\hat{z}^\mu < \kappa, \\ 0, & \text{else}, \end{cases} \tag{30}$$

The second set of weights, meanwhile, is updated using the following rule:

$$\Delta\mathbf{v}^\mu = -\frac{\alpha n_\mu}{N\bar{n}}\mathbf{v}^\mu + \sqrt{2}\frac{\beta n_\mu}{N\bar{n}}\hat{z}^\mu \mathbf{y}^\mu. \tag{31}$$

This Hebbian update rule defines an N-dimensional Ornstein-Uhlenbeck stochastic process with time-dependent coefficients. The evolution of the probability distribution $p(\mathbf{v}, \Delta t)$ is given by the master equation:

$$p(\mathbf{v}, t + \Delta t) = \int d\Delta\mathbf{v} p(\Delta\mathbf{v}|\mathbf{v} - \Delta\mathbf{v}) p(\mathbf{v} - \Delta\mathbf{v}, t), \tag{32}$$

where

$$p(\Delta\mathbf{v}|\mathbf{v} - \Delta\mathbf{v}) = \mathcal{N}\left(-\frac{\alpha n(t)}{N\bar{n}}(\mathbf{v} - \Delta\mathbf{v}), 2\left(\frac{\beta n(t)}{N\bar{n}}\right)^2\right). \tag{33}$$

We can further note that, since the components of $\mathbf{v}$ are not coupled to one another in (31), we can, without loss of generality, consider the evolution of just a single component v_i. In this case the two sides of (32) can

be expanded to obtain

$$p(v_i,t) + \Delta t \frac{\partial p(v_i,t)}{\partial t} + O(\Delta t^2) = p(v_i,t) - \frac{\partial p(v_i,t)}{\partial v_i} \int d\Delta v_i p(\Delta v_i | v_i - \Delta v_i) \Delta v_i$$
$$+ \frac{1}{2} \frac{\partial^2}{\partial v_i^2} p(v_i,t) \int d\Delta v_i p(\Delta v_i | v_i - \Delta v_i)(\Delta v_i)^2 + O((\Delta v_i)^3). \tag{34}$$

Then, using (33) to obtain $\langle \Delta v_i \rangle$ and $\langle (\Delta v_i)^2 \rangle$, (34) leads to the Fokker-Planck equation, which describes the evolution of the probability distribution $p(\mathbf{v})$ as new patterns are learned:

$$\frac{dp(\mathbf{v})}{dt} = \frac{\alpha n(t)}{\bar{n}} \sum_i \frac{\partial}{\partial v_i}[v_i p(\mathbf{v})] + \frac{(\beta n(t)/\bar{n})^2}{N} \sum_i \frac{\partial^2 p(\mathbf{v})}{\partial v_i^2}. \tag{35}$$

In this equation, we have taken the continuous-time limit by letting $\Delta t = 1/N$ and $t \equiv (\mu - \nu)/N$, where $\mu > \nu$, the number of repetitions to be $n(t) \equiv n_\mu$, and the initial condition to be given by the distribution (to be calculated below) $p(\mathbf{v}^\nu)$.

The Fokker-Planck equation (35) can be solved using the Fourier transform (Risken, 1996)

$$p(\mathbf{k}) = \int d\mathbf{v} e^{-i\mathbf{k}\cdot\mathbf{v}} p(\mathbf{v}). \tag{36}$$

With this, (35) becomes

$$\frac{dp(\mathbf{k})}{dt} = -\frac{\alpha n(t)}{\bar{n}} \mathbf{k} \cdot \nabla_{\mathbf{k}} p(\mathbf{k}) - \frac{(\beta n(t)/\bar{n})^2}{N} |\mathbf{k}|^2 p(\mathbf{k}). \tag{37}$$

This equation can be solved by making the following ansatz:

$$p(\mathbf{k}) = \exp\left(-i\mathbf{k}\cdot\mathbf{m}(t) - \frac{1}{2}\mathbf{k}\cdot\mathbf{S}(t)\cdot\mathbf{k}\right), \tag{38}$$

which, by substituting into (37) and requiring that the terms at each order in $\mathbf{k}$ vanish, leads to the following equations for the time-dependent coefficients:

$$\begin{aligned} \frac{dm_i}{dt} &= -\frac{\alpha n(t)}{\bar{n}} m_i \\ \frac{dS_{ij}}{dt} &= -2\frac{\alpha n(t)}{\bar{n}} S_{ij} + 2\frac{(\beta n(t)/\bar{n})^2}{N}\delta_{ij}. \end{aligned} \tag{39}$$

These equations then have the solutions

$$\begin{aligned} m_i(t) &= m_i(0) \exp\left(-\frac{\alpha}{\bar{n}} \int_0^t dt' n(t')\right) \\ S_{ij}(t) &= 2\delta_{ij} \frac{\beta^2}{N\bar{n}^2} \int_0^t dt' \exp\left(-2\frac{\alpha}{\bar{n}} \int_{t'}^t dt'' n(t'')\right) n^2(t-t') \end{aligned} \tag{40}$$

With this, and letting $t = (P - \mu)/N$, we can take the inverse Fourier transform of (38) to obtain the distribution of the final weight vector given the weights at pattern ν:

$$p(\mathbf{v}^P | \mathbf{v}^\nu) = \frac{1}{(2\pi\sigma_P^2/N)^{N/2}} \exp\left(-\frac{N}{2\sigma_P^2}[\mathbf{v}^P - \rho\mathbf{v}^\nu]^2\right), \tag{41}$$

where we have identified $S_{ij}(t) = \sigma_P^2(P,\nu)\delta_{ij}$, with

$$\sigma_P^2(P,\nu) \equiv \frac{2\beta^2}{\bar{n}^2 N} \sum_{\mu=\nu}^{P} \rho^2(P,\mu) n_\mu^2, \tag{42}$$

and we have also identified $m_i(t) = v_i^\nu \rho(P,\nu)$, with

$$\rho(P,\nu) \equiv \exp\left(-\frac{\alpha}{N\bar{n}}\sum_{\mu=\nu}^{P} n_\mu\right). \tag{43}$$

In what follows below, in order to keep expressions compact, we shall write $\sigma_P^2 = \sigma_P^2(P,\nu)$ and $\rho = \rho(P,\nu)$. Further, though it is not strictly necessary, these expressions can be considerably simplified if we make the simplifying assumption that the average of n_μ in (43) over the last $P-\nu$ patterns is equal to its average $\bar{n}$ over the full set of P patterns (or, in the case of (42), that n_μ^2 can be replaced by $\bar{n}^2$). In this case, (43) becomes $\rho(P,\nu) = e^{-\alpha(P-\nu)/N}$, while (42), after performing the summation, becomes $\sigma_P^2 = \beta^2(1-\rho^2)/\alpha$.

Returning to the distribution (41), we can see that it begins as a δ function at $\mathbf{v}^\nu$ and, as more patterns are introduced and $\rho \to 0$, evolves to the following steady-state distribution:

$$p(\mathbf{v}) = \frac{1}{(2\pi\beta^2/\alpha N)^{N/2}} e^{-\alpha N \mathbf{v}^2/2\beta^2}. \tag{44}$$

This is the distribution from which $\mathbf{v}^\nu$ will be drawn in order to calculate the error rate for pattern ν below.

In addition to driving a drift-diffusion process for the weights $\mathbf{v}$, the updates to $\mathbf{v}$ also affect the evolution of the distribution of weights $\mathbf{w}$ due to the appearance of $\mathbf{v}$ in the update rule (30). In order to account for this change, the first two moments of the update $\Delta\mathbf{w}$ must be reevaluated as in (8)-(9), but now averaging over the random variable $\mathbf{y}$ in addition to averaging over $\mathbf{x}$. Because $\langle y_i \rangle = 0$, the first moment in (8) remains unchanged. As for the second moment, (9) generalizes to

$$\begin{aligned}\Sigma_{ij}(\mathbf{w},\mathbf{v}) &\equiv \langle \Delta w_i \Delta w_j \rangle_{\mathbf{x},\mathbf{y}} - \mu_i(\mathbf{w})\mu_j(\mathbf{w}) \\ &= \frac{1}{N^2}\int d\mathbf{x}\, p(\mathbf{x}) \int d\mathbf{y}\, p(\mathbf{y})(1-\mathbf{w}\cdot\mathbf{x}-\mathbf{v}\cdot\mathbf{y})^2 x_i x_j - \mu_i(\mathbf{w})\mu_j(\mathbf{w}) \\ &= \frac{2}{N^2}\left(\left[g(|\mathbf{w}|) + \frac{|\mathbf{v}|^2}{2}\right]\delta_{ij} + h(|\mathbf{w}|) w_i w_j\right).\end{aligned} \tag{45}$$

As before, we will assume that $|\mathbf{w}|^2$ and $|\mathbf{v}|^2$ can be replaced by their average values in the $N \to \infty$ limit. Noting from (44) that $\langle \mathbf{v}^2 \rangle = \beta^2/\alpha$, we have the diffusion tensor $\Sigma_{ij} = 2[\tilde{g}\delta_{ij} + \hat{h} w_i w_j]/N^2$, where $\tilde{g} \equiv g(\hat{w}) + \beta^2/2\alpha$, where, as before, $\hat{w} \equiv \sqrt{\langle |\mathbf{w}|^2 \rangle}$ is taken from numerical simulations. From this result, we see that the equations (18)-(19) determining the evolution of $\mathbf{w}$ can also be applied in the two-pathway case by making the substitution $g \to \tilde{g}$.

As in the previous section, our goal is to calculate the probability of incorrect classification when testing pattern ν after training P patterns in sequence. Including the second input pathway, this quantity is given by

$$\begin{aligned}p(z^\nu \neq \hat{z}^\nu) = &\int d\mathbf{x}^\nu p(\mathbf{x}^\nu) \int d\mathbf{y}^\nu p(\mathbf{y}^\nu) \int d\mathbf{w}^\nu p(\mathbf{w}^\nu) \int d\mathbf{v}^\nu p(\mathbf{v}^\nu) \int d\mathbf{v}^P p\left(\mathbf{v}^P \middle| \mathbf{v}^\nu - \frac{\alpha n_\nu}{N\bar{n}}\mathbf{v}^\nu + \sqrt{2}\frac{\beta n_\nu}{N\bar{n}}\mathbf{y}^\nu\right) \\ &\times \int d\mathbf{w}^P \Theta(-\mathbf{w}^P\cdot\mathbf{x}^\nu - \mathbf{v}^P\cdot\mathbf{y}^\nu) \\ &\times \big[\Theta(1-\mathbf{w}^\nu\cdot\mathbf{x}^\nu - \mathbf{v}^\nu\cdot\mathbf{y}^\nu) p(\mathbf{w}^P|\mathbf{w}^\nu + [1-\mathbf{w}^\nu\cdot\mathbf{x}^\nu - \mathbf{v}^\nu\cdot\mathbf{y}^\nu]\mathbf{x}^\nu/N) \\ &\qquad + \Theta(\mathbf{w}^\nu\cdot\mathbf{x}^\nu + \mathbf{v}^\nu\cdot\mathbf{x}^\nu - 1) p(\mathbf{w}^P|\mathbf{w}^\nu)\big].\end{aligned} \tag{46}$$

As before, we have absorbed all $\hat{z}^\mu$ into the definition of the input activity vectors $\mathbf{x}^\mu$ and $\mathbf{y}^\mu$ by letting $\mathbf{x}^\mu \to \hat{z}^\mu \mathbf{x}^\mu$ and $\mathbf{y}^\mu \to \hat{z}^\mu \mathbf{y}^\mu$, effectively setting all $\hat{z}^\mu = 1$. The first term in the integrand corresponds to cases in which the classification of pattern ν is initially incorrect, so that the weights $\mathbf{w}$ and $\mathbf{v}$ are both updated. The second term corresponds to cases in which the initial classification is correct, so that only the

weights $\mathbf{v}$ are updated. In both cases, the weight distributions for $\mathbf{w}$ and $\mathbf{v}$ evolve according to drift-diffusion processes, as new patterns are learned up until pattern P, at which time, the classification of pattern ν is tested using the final weights $\mathbf{w}^P$ and $\mathbf{v}^P$ (the first Θ function appearing in the integrand).

Again using the trick (21) to represent the Heaviside step functions, the two terms in (46) can be written as

$$p(z^\nu \neq \hat{z}^\nu) = J_1 + J_2, \tag{47}$$

where

$$\begin{aligned} J_1 = &\int_0^\infty du \int_0^\infty du' \int \frac{dv}{2\pi} \int \frac{dv'}{2\pi} e^{i[vu+v'(u'-1)]} \int d\mathbf{x}\; p(\mathbf{x}) \int d\mathbf{y}\; p(\mathbf{y}) \\ &\times \int d\mathbf{w}^\nu p^*(\mathbf{w}^\nu) \int d\mathbf{v}^\nu p^*(\mathbf{v}^\nu) \int d\mathbf{v}^P p\left(\mathbf{v}^P \middle| \mathbf{v}^\nu - \frac{\alpha n_\nu}{N\bar{n}}\mathbf{v}^\nu + \sqrt{2}\frac{\beta n_\nu}{N\bar{n}}\mathbf{y}\right) \\ &\times \int d\mathbf{w}^P p(\mathbf{w}^P | \mathbf{w}^\nu + [1 - \mathbf{w}^\nu \cdot \mathbf{x}^\nu - \mathbf{v}^\nu \cdot \mathbf{y}^\nu]\mathbf{x}^\nu/N) e^{i[(v\mathbf{w}^P + v'\mathbf{w}^\nu)\cdot\mathbf{x} + (v\mathbf{v}^P + v'\mathbf{v}^\nu)\cdot\mathbf{y}]} \end{aligned} \tag{48}$$

and

$$\begin{aligned} J_2 = &\int_0^\infty du \int_0^\infty du' \int \frac{dv}{2\pi} \int \frac{dv'}{2\pi} e^{i[vu+v'(u'+1)]} \int d\mathbf{x}\; p(\mathbf{x}) \int d\mathbf{y}\; p(\mathbf{y}) \\ &\times \int d\mathbf{w}^\nu p^*(\mathbf{w}^\nu) \int d\mathbf{v}^\nu p^*(\mathbf{v}^\nu) \int d\mathbf{v}^P p\left(\mathbf{v}^P \middle| \mathbf{v}^\nu - \frac{\alpha n_\nu}{N\bar{n}}\mathbf{v}^\nu + \sqrt{2}\frac{\beta n_\nu}{N\bar{n}}\mathbf{y}\right) \\ &\times \int d\mathbf{w}^P p(\mathbf{w}^P | \mathbf{w}^\nu) e^{i[(v\mathbf{w}^P - v'\mathbf{w}^\nu)\cdot\mathbf{x} + (v\mathbf{v}^P - v'\mathbf{v}^\nu)\cdot\mathbf{y}]}. \end{aligned} \tag{49}$$

Beginning with J_1, we can shift the integration variable $\mathbf{w}^P \to \mathbf{w}^P - \gamma(\mathbf{v}^\nu \cdot \mathbf{y})\mathbf{x}/N$ and use $\mathbf{x}^2 = N$ in the $N \to \infty$ limit to obtain

$$\begin{aligned} J_1 \overset{(N\to\infty)}{=} &\int_0^\infty du \int_0^\infty du' \int \frac{dv}{2\pi} \int \frac{dv'}{2\pi} e^{i[vu+v'(u'-1)]} \int d\mathbf{x}\; p(\mathbf{x}) \int d\mathbf{w}^\nu p^*(\mathbf{w}^\nu) \\ &\times \int d\mathbf{w}^P p(\mathbf{w}^P | \mathbf{w}^\nu + [1 - \mathbf{w}^\nu \cdot \mathbf{x}]\mathbf{x}/N) e^{i(v\mathbf{w}^P + v'\mathbf{w}^\nu)\cdot\mathbf{x}} K_1(v, v'), \end{aligned} \tag{50}$$

where

$$\begin{aligned} K_1(v, v') &\equiv \int d\mathbf{y}\; p(\mathbf{y}) \int d\mathbf{v}^\nu p^*(\mathbf{v}^\nu) \int d\mathbf{v}^P p\left(\mathbf{v}^P \middle| \mathbf{v}^\nu + \sqrt{2}\frac{\beta n_\nu}{N\bar{n}}\mathbf{y}\right) e^{i[v\mathbf{v}^P + (v' - \gamma v)\mathbf{v}^\nu]\cdot\mathbf{y}} \\ &= \int \frac{d\mathbf{y}}{(2\pi)^{N/2}} e^{-\mathbf{y}^2/2} \int \frac{d\mathbf{v}^\nu}{(2\pi\beta^2/\alpha N)^{N/2}} e^{-\alpha N|\mathbf{v}^\nu|^2/2\beta^2} \\ &\quad \times \int \frac{d\mathbf{v}^P}{(2\pi\sigma_P^2/N)^{N/2}} \exp\left(-\frac{N}{2\sigma_P^2}\left[\mathbf{v}^P - \rho\left(\mathbf{v}^\nu + \sqrt{2}\frac{\beta n_\nu}{N\bar{n}}\mathbf{y}\right)\right]^2\right) e^{i[v\mathbf{v}^P + (v' - \gamma v)\mathbf{v}^\nu]\cdot\mathbf{y}} \\ &= \int \frac{d\mathbf{y}}{(2\pi)^{N/2}} e^{-\mathbf{y}^2/2} \int \frac{d\mathbf{v}^\nu}{(2\pi\beta^2/\alpha N)^{N/2}} \int \frac{d\mathbf{v}^P}{(2\pi\sigma_P^2/N)^{N/2}} \\ &\quad \times \exp\left(-\frac{\alpha N}{2\beta^2}|\mathbf{v}^\nu|^2 - \frac{N}{2\sigma_P^2}|\mathbf{v}^P|^2 + i\mathbf{y}\cdot\left[v\mathbf{v}^P + (v' + (\rho - \gamma)v)\mathbf{v}^\nu + \sqrt{2}\frac{\rho\beta n_\nu}{N\bar{n}}v\mathbf{y}\right]\right) \\ &= \int \frac{d\mathbf{y}}{(2\pi)^{N/2}} \exp\left(-\frac{\mathbf{y}^2}{2}\left[1 + \frac{1}{N}\left(\sigma_P^2 v^2 + \frac{\beta^2}{\alpha}(v' + (\rho - \gamma)v)^2 - 2\sqrt{2}i\frac{\rho\beta n_\nu}{\bar{n}}v\right)\right]\right) \\ &= \left[1 + \frac{1}{N}\left(\sigma_P^2 v^2 + \frac{\beta^2}{\alpha}(v' + (\rho - \gamma)v)^2 - 2\sqrt{2}i\frac{\rho\beta n_\nu}{\bar{n}}v\right)\right]^{-N/2} \\ &\overset{(N\to\infty)}{=} \exp\left(-\frac{\sigma_P^2}{2}v^2 - \frac{\beta^2}{2\alpha}[v' + (\rho - \gamma)v)]^2 + \sqrt{2}i\frac{\beta\rho n_\nu}{\bar{n}}v\right) \end{aligned} \tag{51}$$

Where the Hebbian decay term $\sim \alpha \mathbf{v}^\nu/N$ was dropped in the first line because it vanishes as $N \to \infty$, and the integration variable $\mathbf{v}^P$ was shifted in the third line. Using this result, and noting that the integrals over $\mathbf{x}$, $\mathbf{w}^\nu$, and $\mathbf{w}^P$ are the same as those appearing in (23) (with $\hat{g} \to \tilde{g}$), (50) becomes

$$\begin{aligned} J_1 = &\int_0^\infty du \int_0^\infty du' \int \frac{dv}{2\pi} \int \frac{dv'}{2\pi} \exp\Bigg(-\frac{v^2}{2}\left[(1-\gamma^2)\tilde{g} + \sigma_P^2 + (\rho-\gamma)^2\frac{\beta^2}{\alpha}\right] - \frac{v'^2}{2}\left[\tilde{g} + \frac{\beta^2}{\alpha}\right] \\ &\qquad + iv\left[u + \gamma + \sqrt{2}\frac{\beta\rho n_\nu}{\bar{n}} + i(\rho-\gamma)\frac{\beta^2}{\alpha}v'\right] + iv'[u'-1]\Bigg) \\ = &\frac{1}{\sqrt{8\pi}}\int_{r_1}^\infty dr\ e^{-r^2/2}\mathrm{erfc}\left(\frac{s_1(r)}{\sqrt{2}}\right), \end{aligned} \tag{52}$$

where we have defined

$$r_1 \equiv \frac{\gamma + \sqrt{2}\beta\rho n_\nu/\bar{n}}{\sqrt{(1-\gamma^2)\tilde{g} + \sigma_P^2 + (\rho-\gamma)^2\beta^2/\alpha}} \tag{53}$$

and

$$s_1(r) = \frac{-\sqrt{(1-\gamma^2)\tilde{g} + \sigma_P^2 + (\rho-\gamma)^2\beta^2/\alpha} + r(\gamma-\rho)\beta^2/\alpha}{\sqrt{\left(\tilde{g} + \frac{\beta^2}{\alpha}\right)\left[(1-\gamma^2)\tilde{g} + \sigma_P^2 + (\rho-\gamma)^2\beta^2/\alpha\right] - (\rho-\gamma)^2\frac{\beta^4}{\alpha^2}}}. \tag{54}$$

As in the case without the Hebbian pathway, the last remaining integral in (52) must be performed numerically.

Equation (49) for J_2 can be evaluated in a similar manner. To begin, we express it as

$$\begin{aligned} J_2 \overset{(N\to\infty)}{=} &\int_0^\infty du \int_0^\infty du' \int \frac{dv}{2\pi} \int \frac{dv'}{2\pi} e^{i[vu + v'(u'+1)]} \int d\mathbf{x}\ p(\mathbf{x}) \int d\mathbf{w}^\nu p^*(\mathbf{w}^\nu) \\ &\times \int d\mathbf{w}^P p\left(\mathbf{w}^P|\mathbf{w}^\nu\right) e^{i(v\mathbf{w}^P - v'\mathbf{w}^\nu)\cdot\mathbf{x}} K_2(v,v'), \end{aligned} \tag{55}$$

where

$$\begin{aligned} K_2(v,v') &\equiv \int d\mathbf{y}\ p(\mathbf{y}) \int d\mathbf{v}^\nu p^*(\mathbf{v}^\nu) \int d\mathbf{v}^P p\left(\mathbf{v}^P \middle| \mathbf{v}^\nu + \sqrt{2}\frac{\beta n_\nu}{N\bar{n}}\mathbf{y}\right) e^{i(v\mathbf{v}^P - v'\mathbf{v}^\nu)\cdot\mathbf{y}} \\ &= \int \frac{d\mathbf{y}}{(2\pi)^{N/2}} e^{-\mathbf{y}^2/2} \int \frac{d\mathbf{v}^\nu}{(2\pi\beta^2/N\alpha)^{N/2}} e^{-\alpha N|\mathbf{v}^\nu|^2/2\beta^2} \\ &\quad \times \int \frac{d\mathbf{v}^P}{(2\pi\sigma_P^2/N)^{N/2}} \exp\left(-\frac{N}{2\sigma_P^2}\left[\mathbf{v}^P - \rho\left(\mathbf{v}^\nu + \sqrt{2}\frac{\beta n_\nu}{N\bar{n}}\mathbf{y}\right)\right]^2\right) e^{i(v\mathbf{v}^P - v'\mathbf{v}^\nu)\cdot\mathbf{y}} \\ &= \int \frac{d\mathbf{y}}{(2\pi)^{N/2}} \exp\left(-\frac{\mathbf{y}^2}{2}\left[1 + \frac{1}{N}\left(\sigma_P^2 v^2 + \frac{\beta^2}{\alpha}(\rho v - v')^2 - 2\sqrt{2}i\frac{\rho\beta n_\nu}{\bar{n}}v\right)\right]\right) \\ &= \left[1 + \frac{1}{N}\left(\sigma_P^2 v^2 + \frac{\beta^2}{\alpha}(\rho v - v')^2 - 2\sqrt{2}i\frac{\rho\beta n_\nu}{\bar{n}}v\right)\right]^{-N/2} \\ &\overset{(N\to\infty)}{=} \exp\left(-\frac{\sigma_P^2}{2}v^2 - \frac{\beta^2}{2\alpha}(\rho v - v')^2 + \sqrt{2}i\frac{\rho\beta n_\nu}{\bar{n}}v\right) \end{aligned} \tag{56}$$

Using this result, and noting that the integrals over $\mathbf{x}$, $\mathbf{w}^\nu$, and $\mathbf{w}^P$ are the same as those appearing in (24) (with $\hat{g} \to \tilde{g}$), (55) becomes

$$\begin{aligned} J_2 = &\int_0^\infty du \int_0^\infty du' \int \frac{dv}{2\pi} \int \frac{dv'}{2\pi} \exp\left(i[uv + (u'+1)v'] - \frac{\tilde{g}}{2}[v^2 + v'^2 - 2\gamma v v']\right) \\ &\times \exp\left(-\frac{\sigma_P^2}{2}v^2 - \frac{\beta^2}{2\alpha}(\rho v - v')^2 + \sqrt{2}i\frac{\rho\beta n_\nu}{\bar{n}}v\right) \\ = &\frac{1}{\sqrt{8\pi}}\int_{r_2}^\infty dr\ e^{-r^2/2}\mathrm{erfc}\left(\frac{s_2(r)}{\sqrt{2}}\right), \end{aligned} \tag{57}$$

where we have defined

$$r_2 = \frac{\sqrt{2}\rho\beta n_\nu/\bar{n}}{\sqrt{\tilde{g} + \sigma_P^2 + \rho^2\beta^2/\alpha}} \tag{58}$$

and

$$s_2(r) = \frac{\sqrt{\tilde{g} + \sigma_P^2 + \rho^2\beta^2/\alpha} + (\gamma\tilde{g} + \rho\beta^2/\alpha)r}{\sqrt{\left(\tilde{g} + \frac{\beta^2}{\alpha}\right)(\tilde{g} + \sigma_P^2 + \rho^2\beta^2/\alpha) - (\gamma\tilde{g} + \rho\beta^2/\alpha)^2}}. \tag{59}$$

It is straightforward to check that, in the limit $\beta \to 0$, which corresponds to shutting off the Hebbian input pathway, we recover $J_{1,2} \to I_{1,2}$ from the simple perceptron result.

Finally, we can calculate the probability of making a weight update in a given step, which in the two-pathway case is given by

$$\begin{aligned} q &= \int \frac{d\mathbf{x}}{(2\pi)^{N/2}} e^{-|\mathbf{x}|^2/2} \int \frac{d\mathbf{y}}{(2\pi)^{N/2}} e^{-|\mathbf{y}|^2/2} \int \frac{d\mathbf{w}}{(2\pi\hat{w}^2/N)^{N/2}} e^{-N|\mathbf{w}|^2/2\hat{w}^2} \\ &\quad \times \int \frac{d\mathbf{v}}{(2\pi\beta^2/\alpha N)^{N/2}} e^{-N\alpha|\mathbf{v}|^2/2\beta^2} \Theta(1 - \mathbf{w}\cdot\mathbf{x} - \mathbf{v}\cdot\mathbf{y}) \\ &= \frac{1}{2}\mathrm{erfc}\left(-\frac{1}{\sqrt{2(\hat{w}^2 + \beta^2/\alpha)}}\right). \end{aligned} \tag{60}$$

In the case where $\beta^2/\alpha = 0$, this evaluates to the earlier result $q \simeq 0.798$. For nonzero β, on the other hand, q decreases and approaches 0.5 as $\beta^2/\alpha \to \infty$.

Supplementary Figures

Learning with gradient descent

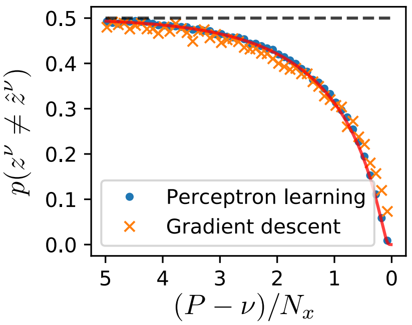

Supplemental Figure 1: Training with gradient descent yields a forgetting curve similar to the perceptron case.

The perceptron learning rule, according to which synaptic weights are adjusted to produce the correct output in a single step, is mathematically convenient but biologically questionable. In order to address this, we simulated the forgetting curve using gradient descent learning, a widely used supervised learning algorithm in which small updates are accumulated over many repetitions to minimize the readout error.

In this case the output of the neuron is $z_a^\mu = \text{sgn}(\mathbf{w}^\mu \cdot \mathbf{x}^\mu + \sigma\xi_a)$, where $\xi_a \sim \mathcal{N}(0,1)$ is drawn randomly for step a. The number of steps for each pattern was chosen to be $N_{\text{steps}} = 100$, and the learning rate $\eta = 0.01$ and the noise amplitude $\sigma = 0.2$ were chosen by grid search to maximize the area between the forgetting curve and chance performance. The noise is not strictly necessary in gradient descent learning, but is included here so that a finite classification margin will be obtained, as in the case of the perceptron learning rule (Figure 1b). In this case, the synaptic weights were updated according to the gradient-descent update rule $\Delta w_i^\mu = \sum_{a=1}^{N_{\text{steps}}} \delta w_{i,a}^\mu$, where

$$\delta w_{i,a}^\mu = \frac{\eta}{N_x}[\hat{z}^\mu - z_a^\mu]x_i^\mu.$$

As shown in Supplemental Figure 1, the forgetting curve obtained for the perceptron with this alternative learning rule is similar to that obtained using the perceptron learning rule.

Two-pathway forgetting curves depend on Hebbian learning and decay rates

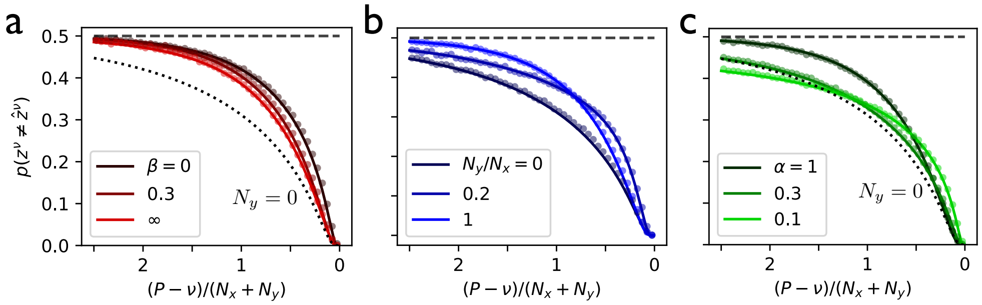

Supplemental Figure 2: Forgetting curves in the two-pathway model with each pattern trained once. **(a)** The forgetting curve for different values of the Hebbian learning rate β, with $\alpha = 1$ and $N_x = N_y$ (dotted line shows the case with no second pathway). **(b)** The forgetting curve for different values of N_y/N_x, with $\alpha = \beta = 1$. **(c)** The forgetting curve for different values of the Hebbian weight decay rate α, with $\beta = 1$ and $N_x = N_y$. In (b)-(d), solid lines are theoretical results; points are simulations with $N_x = N_y = 1000$.

In the two-pathway model, we kept $n_\nu = \bar{n}$ constant for all patterns and investigated the dependence of the forgetting curve (4) on its other parameters. Starting with the Hebbian learning rate, we found that nonzero values of β shifted the forgetting curve slightly downward, modestly reducing the error rate for all patterns (Supplemental Figure 2a). Whether this qualifies as a true improvement, however, depends somewhat on bookkeeping. For a fixed total number of synapses $N_x + N_y$, the error rate can be reduced by allowing for Hebbian learning. However, the error rate is reduced even more by eliminating the Hebbian synapses entirely (dotted curve in Supplemental Figure 2a), which decreases the denominator $N_x + N_y$ by setting $N_y = 0$. Stated differently, if the goal is to minimize the error rate for a fixed total number of synapses, this is accomplished most effectively by letting all of the synapses be updated with supervised learning rather than with Hebbian learning (Supplemental Figure 2b). If, on the other hand, the goal is to minimize the error rate for a fixed number N_x of supervised synapses, then a benefit is obtained from including additional synapses with Hebbian learning. The exception to these conclusions occurs for small values of the Hebbian decay rate α, in which case very old memories can persist for longer with error rates below chance level (Supplemental Figure 2c). In this case, there is a benefit to adding Hebbian synapses even if they are counted against the total $N_x + N_y$.

Optimal forgetting rate and memory capacity

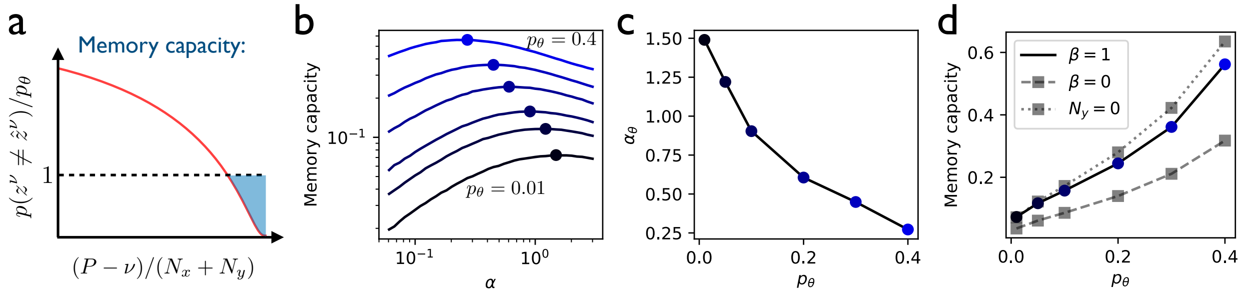

Supplemental Figure 3: Error tolerance determines the optimal forgetting rate and memory capacity. **(a)** Given a threshold p_θ of incorrect classification probability, the memory capacity is defined as the area above the forgetting curve and below the threshold, normalized by p_θ. **(b)** For each value of p_θ, the memory capacity is optimized with respect to the Hebbian forgetting rate α. (For all curves, $\beta = 1$ is fixed, $N_x = N_y$, and $n_\nu = \bar{n}$ for all patterns.) **(c)** The optimal forgetting rate from (b) as a function of p_θ. **(d)** The memory capacity from (b) as a function of the classification threshold p_θ. Dashed line shows the capacity with a second pathway with no learning ($N_y = N_x$, $\beta = 0$); Dotted line shows the capacity with no second pathway ($N_y = 0$).

We investigated the effects of the parameters α and β in the two-pathway model, while still holding n^ν constant for all patterns, by setting a threshold p_θ for the acceptable error rate, then defining the memory capacity as the integrated area between the forgetting curve and this threshold value, normalized by p_θ (Supplemental Figure 3a). Because the forgetting curve was found to have relatively weak dependence on β (Figure 2b), we fixed $\beta = 1$ and considered the effects of α and of p_θ on the memory capacity. For a given choice of p_θ, we found the value of α that maximized the memory capacity (Supplemental Figure 3b). This led to the conclusion that, the larger the error tolerance p_θ, the smaller the forgetting rate α should be in order to maximize the memory capacity (Supplemental Figure 3c). Finally, we found that the optimized memory capacity increases as the error tolerance becomes greater (Supplemental Figure 3d). Consistent with our observations from Figure 2, we found that the memory capacity, which is normalized by the total number of synapses $N_x + N_y$, is improved by learning in the second pathway if $N_x + N_y$ is held constant (solid vs. dashed curve in Supplemental Figure 3d), but is even larger if the second pathway is left out entirely (dotted curve in Supplemental Figure 3d), again indicating that adding supervised synapses is a better strategy than adding unsupervised synapses if the goal is to optimize the forgetting curve for a fixed total number of synapses.

Recall performance depends on repetitions and number of repeated patterns

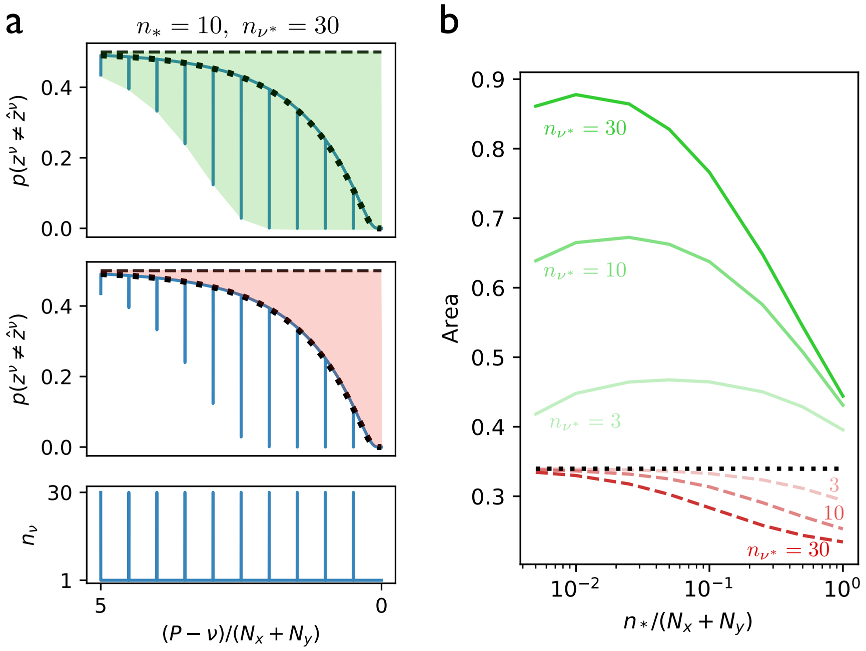

Supplemental Figure 4: Recall performance depends on repetitions and number of repeated patterns. **(a)** In the two-pathway model in which n_* evenly spaced patterns are each repeated n_{ν^*} times during training (bottom panel), the green shaded area (top panel) provides a measure of the recall performance for the repeated patterns, while the red shaded area (middle panel) provides a measure of the recall performance for all other patterns. **(b)** By repeating the subset of patterns n_* times, recall is significantly enhanced for the patterns during training (green curves) while being slightly diminished for the nonrepeated patterns (red curves).

In Supplemental Figure 4, we illustrate the tradeoff in the two-pathway model between the enhancement of recall for patterns that are repeated during training versus the impairment of recall for the nonrepeated patterns. In Supplemental Figure 4a, we define the green shaded area as a metric of how well the repeated patterns are retained, and we define the red shaded area as a metric of how well the non-repeated patterns are retained. (The area between the dashed and dotted lines provides a baseline in which no patterns are repeated multiple times.) As shown in Supplemental Figure 4b, the enhancement for repeated patterns (green curves) is far greater than the impairment for nonrepeated patterns (red curves) if the number of repeated patterns is much less than the total number of inputs (N_x+N_y). However, as the number of repeated patterns becomes comparable to the number of inputs, the impairment for the nonrepeated patterns becomes comparable in magnitude to the enhancement of the repeated patterns.

Forgetting curves decay exponentially

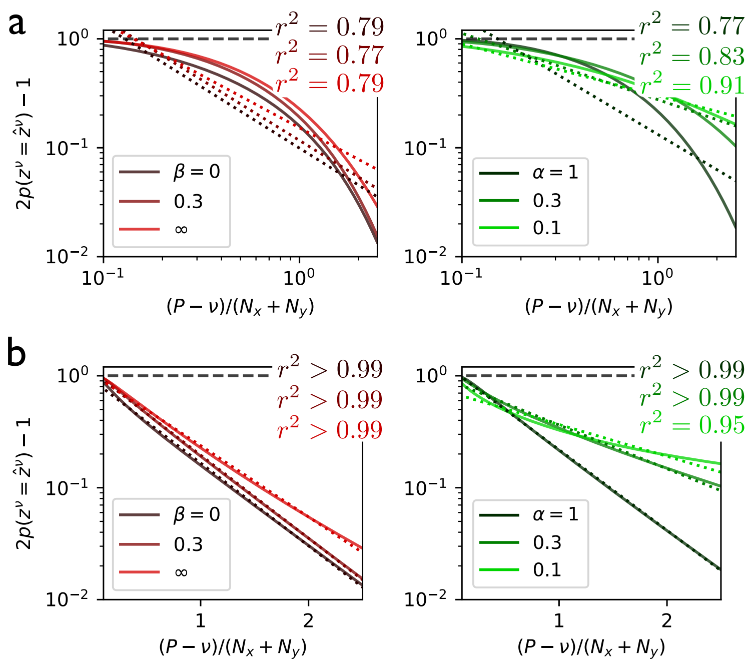

Supplemental Figure 5: Forgetting curves exhibit approximately exponential decay. **(a)** Forgetting curves from Figure 2b (*left*) and 2d (*right*) plotted on logarithmic axes, with $N_x = N$. Dotted lines are best fits with power law decay. **(b)** Forgetting curves as in (a), but plotted with just one logarithmic axis. Dotted lines are best fits with exponential decay.

Many previous studies on memory have shown that forgetting curves are well described by curves decaying as a power law, in which the probability of correct recall has the form $\sim t^{-a}$, where t is time and $a > 0$. Supplemental Figure 5a shows that such a fitting function, which appears as a straight line in a log-log plot, does a relatively poor job of fitting the forgetting curves from the two-pathway model. In contrast, exponentially decaying functions, in which the probability of correct recall $\sim e^{-at}$ and which appear as straight lines on semilogarithmic plots, provide a good fit to the forgetting curves (Supplemental Figure 5b).

Spaced repetition

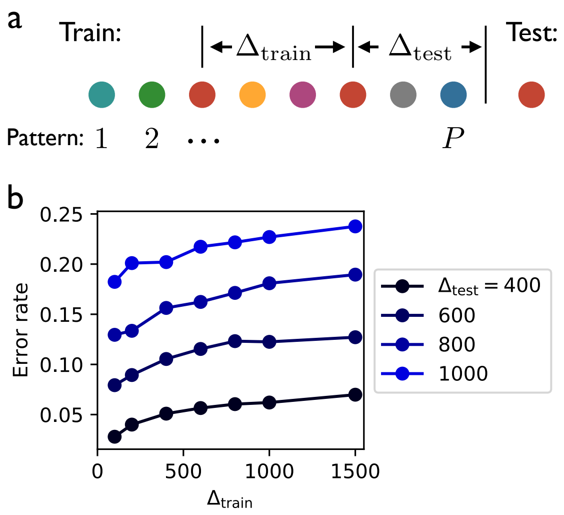

Supplemental Figure 6: Short training intervals during spaced repetition lead to better testing performance. **(a)** The two-pathway network is trained with random sequential patterns, with one particular pattern presented twice. **(b)** For any interval Δ_{test} between the second presentation and the testing phase, the testing performance for the repeated pattern is best when the interval between the presentations during training is short. Results are simulations from a two-pathway network with $N_x = N_y = 1000$ and $\alpha = \beta = 1$.

In simulations of the two-pathway model, the neuron was trained in sequence to perform P classifications. All of the input patterns were distinct, except for a single pattern that was presented twice during training, with an interval Δ_{train} between presentations. The interval between the second presentation and the testing phase was Δ_{test}. For any Δ_{test}, smaller values of Δ_{train} always led to a lower error rate for the repeated pattern during testing (Figure 6b).

In addition, we note that repetition of patterns has no significant effect at all in the single-pathway model without Hebbian weights. In this case, upon the second presentation of the repeated pattern during training, the weight vector will either not be updated at all (if classification is already correct with sufficient margin) or will be updated to lie on the correct side of the classification boundary plus a margin (if the classification is initially incorrect or correct with insufficient margin). In neither of these two cases is there a benefit to having seen the pattern before, since one of these same things would have happened if the first presentation had not occurred.

Thus, while the information accumulated in the Hebbian weights of the two-pathway model is an important ingredient for describing nontrivial effects due to spaced repetition, the single-neuron model appears to be unable to account for the experimentally observed existence of optimal repetition intervals (Glenberg, 1976), and thus leaves room for future work on this topic.

References

[1] H. Risken. *Fokker-Planck Equation*. Springer, 1996.

[2] Stefano Fusi and L.F. Abbott. Limits on the memory storage capacity of bounded synapses. *Nature Neuroscience*, 10(4):485, 2007.

[3] Marcus K Benna and Stefano Fusi. Computational principles of synaptic memory consolidation. *Nature Neuroscience*, 19(12):1697, 2016.

[4] Arthur M Glenberg. Monotonic and nonmonotonic lag effects in paired-associate and recognition memory paradigms. *Journal of Verbal Learning and Verbal Behavior*, 15(1):1-16, 1976.
